# How Do the Morphological Abnormalities of Femoral Head and Neck, Femoral Shaft and Femoral Condyle Affect the Occurrence and Development of Medial Knee Osteoarthritis

**DOI:** 10.1111/os.13910

**Published:** 2023-10-24

**Authors:** Shiqi Qin, Ming Li, Yanfeng Jia, Wei Gao, Juncai Xu, Boxuan Zhang, Hailong Guo, Ao Feng, Ran Sun

**Affiliations:** ^1^ Hebei Medical University Third Affiliated Hospital Shijiazhuang China; ^2^ Third Military Medical University Southwest Hospital Chongqing China; ^3^ Hebei No. 8 People's Hospital Shijiazhuang China

**Keywords:** Alignment, Coronal bowing, Femoral off‐set, Hip‐knee‐ankle angle, Knee osteoarthritis, mLDFA

## Abstract

**Objective:**

At present, the main viewpoint is that tibial varus is the main reason of medial knee osteoarthritis (OA), and high tibial osteotomy (HTO) is also the main alignment correction method to correct medial knee OA. In contrast, the impact of the anatomical alignment of the femur on medial knee OA is often overlooked. We evaluated the increased risk for medial knee OA because a varus alignment could be attributed to the anatomical reasons that include hip anatomy, femoral shaft bowing (FSB) and femoral condylar dysplasia.

**Methods:**

The present research adopted a cross‐sectional study method. We selected 62 patients with HTO in the Third Hospital of Hebei Medical University from June 2021 to March 2022 as the HTO group and 55 healthy volunteers as the control group. Femoral neck‐shaft angle (NSA), lateral FSB, mechanical lateral distal femoral angle (mLDFA) and hip‐knee‐ankle (HKA) was radiographically examined within the two groups. The femoral neck length and offset were also measured, and the ratio is represented by the ratio of the femoral neck length to off‐set (N/O). The 2‐tailed Student *t*‐test was used to compare the differences between groups when the data were in accordance with a normal distribution. Otherwise, the Mann–Whitney *U* tests was used to compare the differences between groups.

**Result:**

Compared to the control group, the HTO group had a higher offset (*p* < 0.05), greater femoral neck length (*p* < 0.05), and decreased (more varus) NSA (*p* < 0.05). The HKA in the HTO group was 172.20 ° (3.50°), which was significantly lower than that of the control group 177.00° (3.05°), (*p* < 0.001), while the medial OA was associated with more varus HKA. The mean mLDFA was 89.10 ° (2.35°) and 87.50° (2.85°) in the HTO and control groups (*p* < 0.005), respectively. The mean lateral FSB values of the full‐length radiographs were larger (*p* < 0.001) in the HTO group (4.24° ± 3.25°) than that in control group (1.16° ± 2.32°).

**Conclusion:**

The reduction of NSA (coxa vara) and the increase of the mLDFA can lead to medial knee OA, while the lateral FSB also affects medial OA. We believe that femoral deformity is also one of the cause of the medial knee OA. Therefore, it is necessary to evaluate the joint deformity of the femur and tibia before surgery in order to determine whether to use HTO alone to correct the lower limb alignment.

## Graphical abstract

We found that the anatomical changes of each segment of femur (the abnormal morphology of the femoral head and neck, the degree of femoral shaft bending, and the abnormal morphology of the femoral condyle) had an important impact on the occurrence and development of medial knee osteoarthritis.

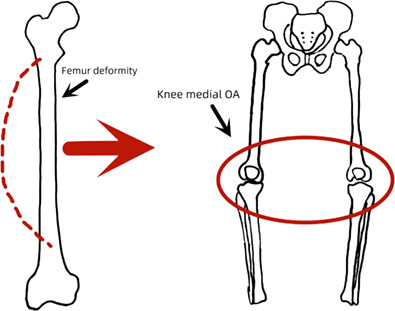

## Introduction

Knee osteoarthritis (KOA), which based on degenerative pathological changes, has been widely studied by scholars all over the world due to its high morbidity and high teratogenicity risks. There are many factors that contribute to the development of KOA, such as genetics, age, obesity, and trauma.^1^, ^2^, ^3^ However, since leg alignment has an significant influence on the knee joint load,^4^ correction of lower limb alignment is an important principle for surgical treatment of KOA.

The exploration of the relationship between leg alignment and KOA has attracted increasing attention of global scholars that have drawn important conclusions. This change in leg alignment might increase the risk for osteoarthritis of the medial knee since a varus alignment increases the knee joint load. Studies have shown that even in healthy neutral knee joints, most activities of daily living will put more load on the medial compartment of the knee than on the lateral compartment, which is also used to explain the finding that medial knee OA is more prevalent than lateral knee OA.^5^, ^6^ The morphological characteristics of the tibia and femur can affect the alignment of the lower limb and thus the progression of osteoarthritis. The current general view suggests that tibial varus is the leading cause of the varus status of the lower limbs, therefore, relevant distances and angles of the tibia have received extensive attention from scholars, and the high tibial osteotomy (HTO) is often used to correct the alignment of the lower limb and treat the medial KOA. However, the abnormal morphological structure of the femur will also affect the alignment of the lower limb. These factors include abnormal morphology of the femoral head and neck, the degree of femoral shaft bending, and the abnormal morphology of the femoral condyle. Some scholars have pointed out that the morphological features of the proximal femur in the coronal plane will affect the morphology of the distal femur in the coronal plane and the transverse plane, and then affects the alignment of the lower limb. The degree of the femoral neck anteversion and the morphological parameters of the proximal femur will affect the morphology of the distal femoral trochlea. However, in presented study, this phenomenon only occurs in females.^7^, ^8^ Suardi *et al*. found that an increased abductor angle and an increased height of hip center were associated with lateral compartment OA.^9^ Moon *et al*. focus on the gender difference of the incidence rate of the lateral KOA. And they explore whether the differences in hip anatomy between male and female are the reason for the above situation.^10^ Mullaji *et al*. use measurement of the alignment of the lower limbs to classify KOA and explore the similarities and differences between different types.^11^ However, the above researchers focused more on the influence of the anatomy of the hip or proximal femur on KOA. Other researchers have measured angles that reflect anatomic abnormalities in the hip and tried to look for correlations with changes in the alignment of lower limb. However, they focused more on the anatomy of the hip, rather than the deformity of different segments of femur that leads to knee varus, and affects the progression of medial KOA.^12^, ^13^, ^14^, ^15^ There is very little research on this aspect and there are many controversies that still remain. Therefore, the purpose of this study was to: (i) investigate the relationship between the data reflecting the morphological structure of the proximal femur and the medial KOA; (ii) to discuss the impact of the femoral shaft deformity on the medial KOA; and (iii) to explore the relationship between the data reflecting the morphological structure of the distal femur and the medial KOA. This data includes the femoral neck‐shaft angle (NSA), femoral neck length, off‐set, the ratio of the femoral neck length to off‐set (N/O), the lateral femoral shaft bowing (FSB), mechanical lateral distal femoral angle (mLDFA), and hip‐knee‐angle angle (HKA). We set patients with KOA as the experimental group and healthy subjects as the control group. The above data were respectively measured in the experimental group and the healthy control group. The differences in the above data results between the two groups were compared and statistical analysis was carried out to draw conclusions. Our hypothesis is that anatomical abnormalities of different segments of the femur play an important role in the occurrence and development of medial KOA. These results warrant our investigation to find out whether undergoing the HTO alone can protect patients from the progression of KOA. The results also have important implications for the preoperative plan design, postoperative prognostic assessment and exercise rehabilitation of performing the HTO or a total knee arthroplasty (TKA) to treat KOA.

## Materials and Methods

### Patients

A total of 62 patients (23 males and 39 females) scheduled to undergo HTO as treatment for medial KOA in the Third Hospital of Hebei Medical University from June 2021 to March 2022 were enrolled. The definition of KOA was based on Kellgren‐Laurence grade > II on anteroposterior standing radiographs, and the HKA should be less than 177°.^16^ Cases meeting the above criteria were classified as the HTO group. Some angles of the knee need to be measured before the surgery, therefore, in this group, the radiographs and CT images were recorded prospectively for the purpose of surgical treatment, with diagnosis of the unilateral symptomatic knee OA confirmed on standard anteroposterior and lateral radiographs. Exclusion criteria were as follows: (i) those with missing radiographs or incomplete radiograph sets, or those which were inappropriate for obtaining accurate measurements; (ii) those with knees with valgus deformity; (iii) patients with osteonecrosis of the knee, hip, or ankle joint, history of knee injury, previous surgery, and previous therapy with estrogen, bisphosphonate, or parathyroid hormone; and (iv) patients whose knee could not be maintained in extension in the standing position, knee flexion deformity >5° and knee hyperextension.^17^

We enrolled 55 healthy volunteers (18 males and 37 females) as controls. In this group, the definition of knee OA was the Kellgren‐Lawrence grade ≤ II on anteroposterior standing radiographs. These patients had no or very mild knee symptoms, and no severe deformity of the knee.

The study was approved by the Hebei Medical University Third Affiliated Hospital Institutional ethics review board. The protocol number: Ke 2022‐112‐1. Approved by the Institutional ethics review board, our research is exempt from the written informed consent.

### Radiographic Assessment

Image acquisition and measurement data of patients and healthy volunteers were conducted in the Third Hospital of Hebei Medical University. Anteroposterior full‐length lower limb radiographs, with the knee joint maintained in extension, were obtained for the patients or the volunteers in the standing position. The lower limbs were positioned so that the patella faced forward, and the x‐ray beam was centered on the knee.

All data measuring was performed using a picture archiving and communication system (PACS, PI View STAR, version 5025; Infnitt, Seoul, South Korea). We define morphological and structural data of the different segments of femur as follows: when measuring the femoral shaft, the proximal end was defined as the part of the femur proximal to the lower border of the lesser trochanter, and the distal end was defined as the junction between the femoral shaft and the condylar region, which was indicated by the flare of the posterior cortex.

**Fig. 1:**
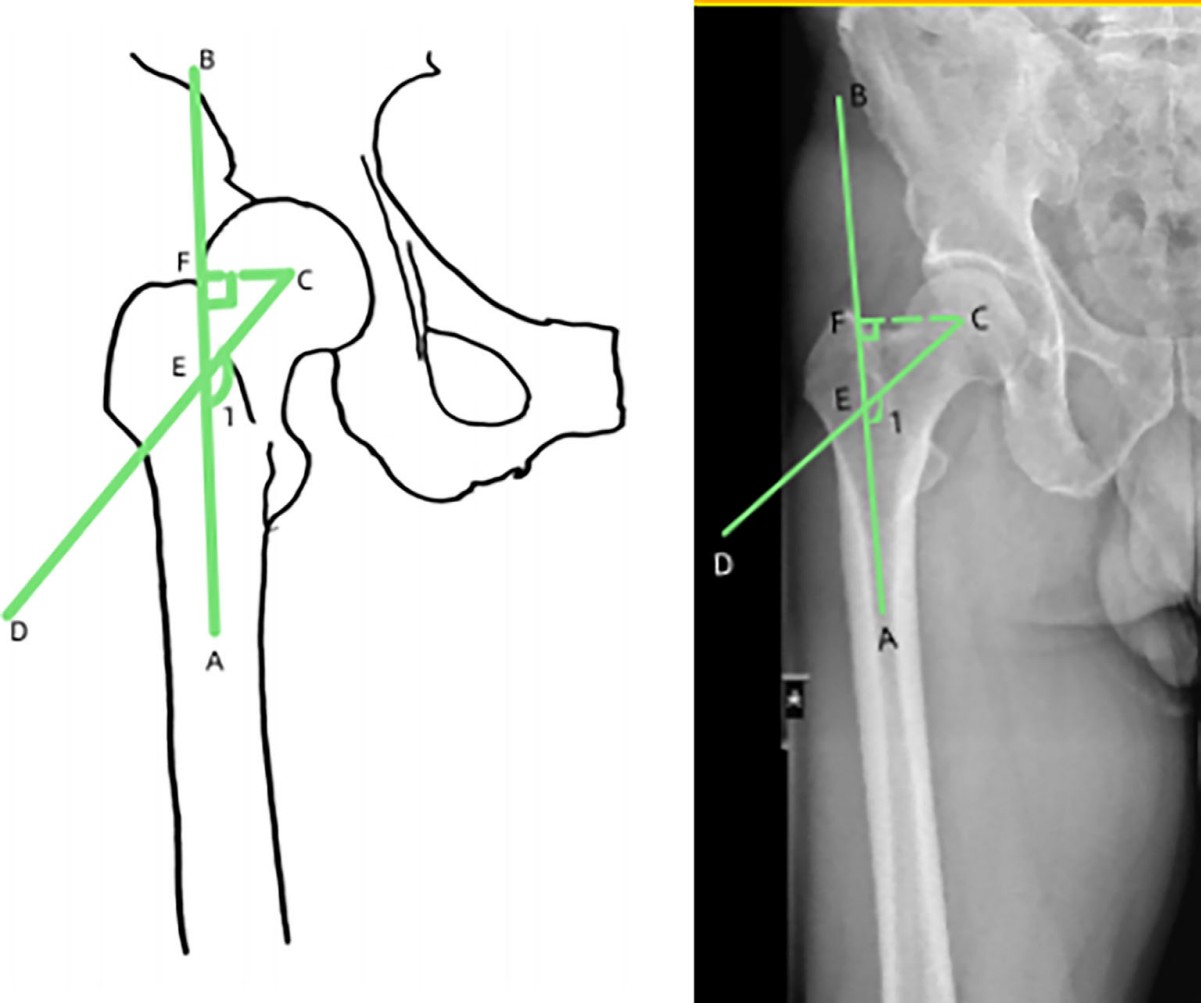
Angle 1: femoral neck‐shaft angle (NSA). CE, femoral neck length; CF, off‐set.

**Fig. 2:**
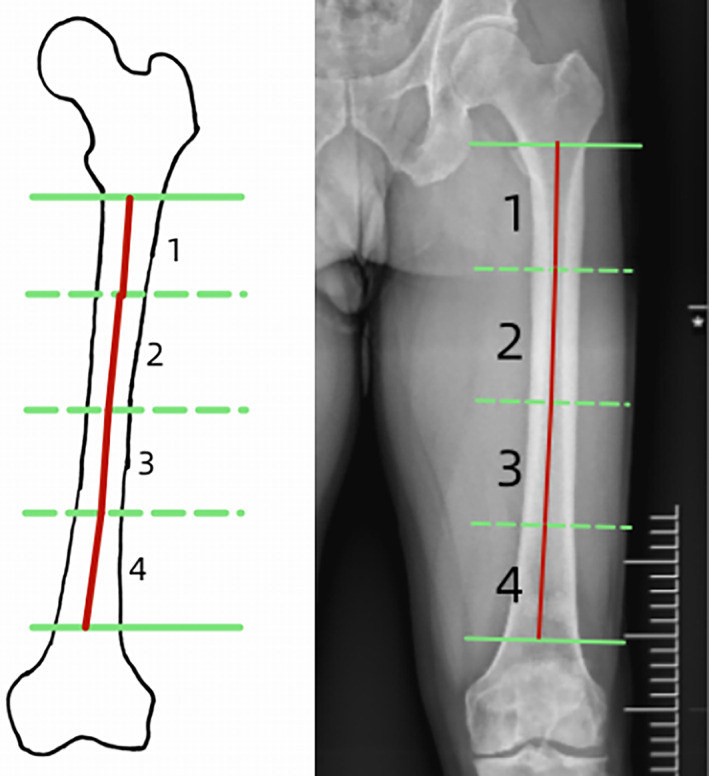
The angle between axis 1 and axis 4: the lateral femoral shaft bowing (FSB).

**Fig. 3:**
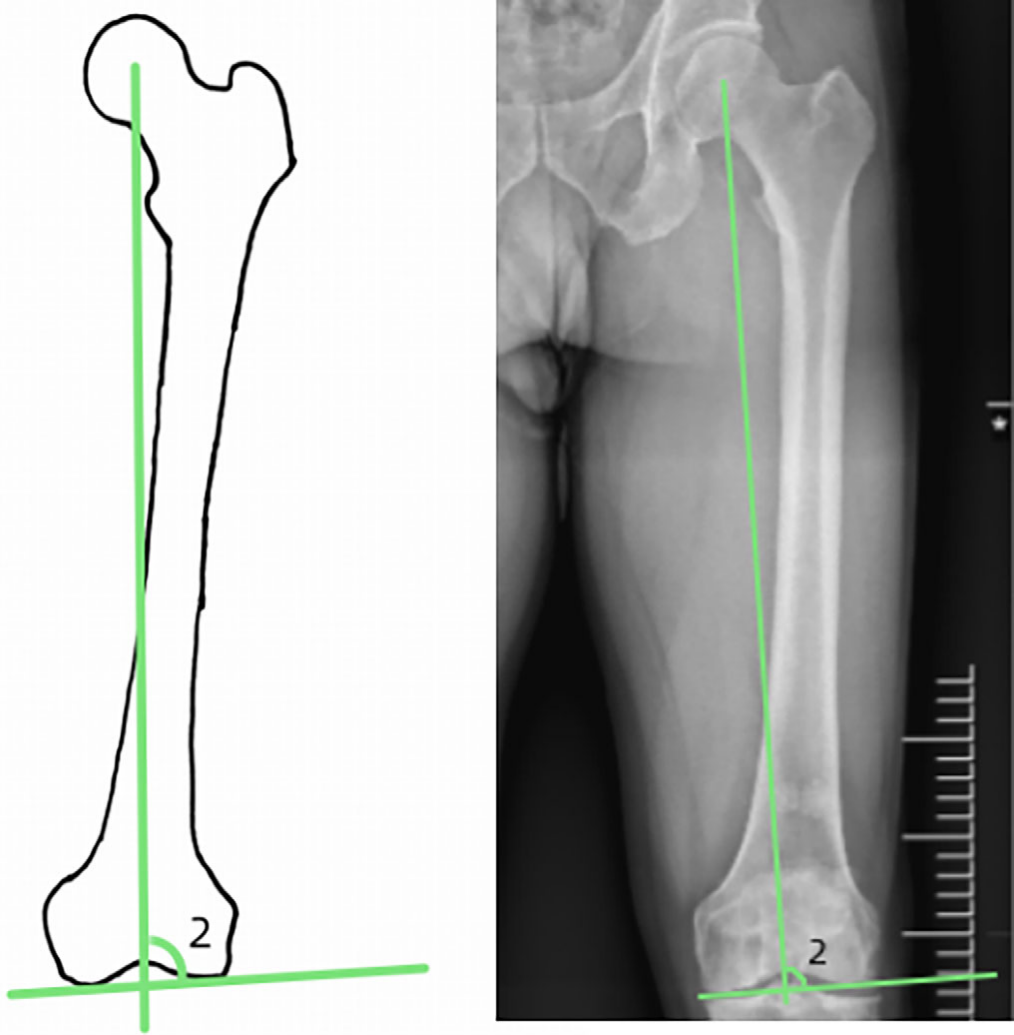
Angle 2: mechanical lateral distal femoral angle (mLDFA).

**Fig. 4:**
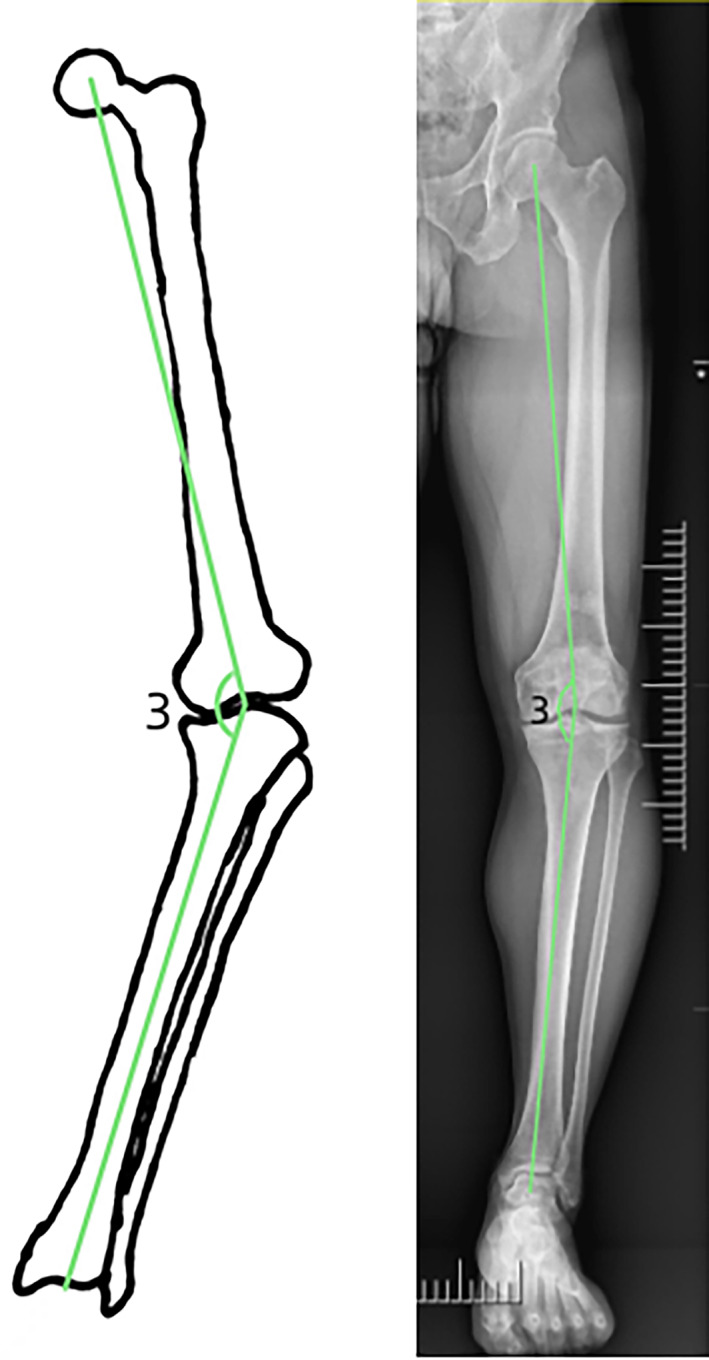
Angle 3: hip‐knee‐angle angle (HKA).

The femoral NSA was the angle of intersection between the femoral neck axis and the proximal femoral shaft axis. The normal value of the NSA ranges from 125° to 135°. Two lines were used to reconstruct the NSA, one in the center of the femoral neck and one in the center of the femoral shaft. The distance between the center of the femoral head and the point where these two lines intersected represented the femoral neck length. The horizontal distance between the femoral head center and the line through the center of the femoral shaft represented the “offset” (Head‐Shaft distance). In order to eliminate individual differences, the femoral neck length and offset were measured, and the ratio was represented by N/O (Fig. 1).^18^, ^19^, ^20^ The coronal bowing of the femur was measured by dividing the femoral diaphysis into four equal parts. A line that best described the midpoint of the endosteal canal was drawn in each quarter. The overall femoral bowing was measured as the angulation between the proximal and distal quarters of the femoral diaphysis (Fig. 2).^21^ Paley's principle was used to name the mLDFA. mLDFA was defined as the lateral angle between the femoral mechanical axis (the line joining the center of the femoral head and the center of the knee joint) tangent to the femoral condyles (Fig. 3).^22^ Normal values for each of the radiological parameters reported in literature are 85° to 90° for mLDFA. The HKA is similar to the mechanical axis and represents the varus/valgus configuration of the knee. The HKA is the angle between the femoral and the tibial mechanical axes (line joining the center of the knee joint and the center of the ankle joint) (Fig. 4).^15^ A value greater than 180° equals a valgus alignment, a value smaller than 180° equals a varus alignment.

### Statistical Analysis

The radiographic parameters of our study were evaluated by two independent investigators who were blinded to the trial interventions. The result were averaged from the two investigators and was used in the analysis.^17^ The Interclass correlation coefficient (ICC) was used to measure inter‐observer reliabilities for the radiographic assessment. The ICC measured to be >0.8 for all measurements.

All statistical analyses were performed on IBM SPSS Statistics, Version 26.0 (IBM Corp., Armonk, NY, USA). After data grouping, the chi‐square test was applied to compare categorical variables. Kolmogorov–Smirnov and Shapiro–Wilk tests were used to test for the normality of data distribution. Mean ± standard deviation (SD) was used to represent measurement data with a normal distribution, and a two‐tailed Student *t*‐test was used to compare the two groups. Mann–Whitney *U* tests were used to compare data between the two groups when the data did not follow a normal distribution, and medians (interquartile ranges) was used to represent measurement data without a normal distribution. Probability values less than 0.05 (two‐tailed) were considered indicative of statistical significance.

## Results

The mean age was 55.24 years (35 to 70) for the HTO group, and 54.22 years (39 to 72) for the control group. The two groups were comparable regarding gender, age, and body mass index without statistically significant differences (Table 1).

**TABLE 1:** Sample characteristics

	HTO group	Control group	*t*/*X* ^2^ value	*p* value
Knees (*n*)	62	55	‐	‐
Sex (female/male)	39/23	37/18	0.244	0.621
Age at surgery (years)	55.24 ± 7.60	54.22 ± 8.22	−0.700	0.485
Body mass index (kg/m^2^)	26.25 ± 2.46	25.73 ± 2.62	−1.088	0.279

Compared to the control group, the HTO group had a lower (more varus) NSA (125.11° ± 5.30°, *p* < 0.05), corresponding to the hip varus. The neck length and off‐set of the HTO group was both longer than in the control group (*p* < 0.05), and the N/O in the HTO group was 1.20 (0.12), which was significantly lower than that of the control group (1.25 [0.11], *p* < 0.05) (Table 2).

**TABLE 2:** The NSA, femoral neck length, offset and N/O value between two groups

	HTO group	Control group	*t*/*Z* value	*p* value
NSA (°)	125.11 ± 5.30	127.49 ± 4.22	2.666	0.009
Neck length (cm)	5.12 ± 0.52	4.93 ± 0.48	−2.073	0.040
Offset (cm)	4.20 ± 0.59	3.92 ± 0.51	−2.810	0.006
N/O value	1.20 (0.12)	1.25 (0.11)	−2.422	0.015

Abbreviations: HTO, high tibial osteotomy; N/O, the ratio of the femoral neck length to off‐set; NSA, neck‐shaft angle.

The HKA in the full‐length radiographs were significantly lower (*p* < 0.001) in the HTO group (172.20° [3.50°]) than in the control group (177.00° [3.05°)], which indicated that the knee varus is associated with the progression of medial KOA. The FSB was significantly higher (*p* < 0.001) in the HTO group (4.24° ± 3.25°) than in the control group (1.16° ± 2.32°), and the mLDFA was also higher in the HTO group (89.10° [2.35°], *p*<0.01) (Table 3).

**TABLE 3:** HKA, mLDFA and lateral FSB between two groups

	HTO group	Control group	*t*/*Z* value	*p* value
HKA (°)	172.20 (3.50)	177.00 (3.05)	−8.048	*p* < 0.001
FSB (°)	4.24 ± 3.25	1.16 ± 2.32	−5.839	*p* < 0.001
mLDFA (°)	89.10 (2.35)	87.50 (2.85)	−3.168	0.002

Abbreviations: FSB, femoral shaft bowing; HKA, hip‐knee‐angle; HTO, high tibial osteotomy; mLDFA, mechanical lateral distal femoral angle.

## Discussion

Through our research, we found that tibial varus is not the only cause of the medial knee OA, the anatomical structure of the proximal femur, femoral shaft and distal femur all have a certain impact on the occurrence of medial KOA. There were significant differences in NSA, FSB and mLDFA between the control group and the HTO group. HTO restores the lower limb force line by changing the shape of the proximal tibia; however, it cannot eliminate other factors causing osteoarthritis. Many studies have found that some patients receiving HTO have abnormal femoral structure (including the hip joint, femoral shaft, and femoral condyle), indicating that the abnormalities of different segments of femur are an important factor leading to medial KOA.^23^, ^24^, ^25^

### Proximal Femur and Knee Osteoarthritis

Many studies have detected a significant relationship between hip geometry and knee OA; however, it remains unclear if the association is due to altered hip biomechanics, an influence on knee alignment or both.^13^, ^14^, ^15^ Some scholars have proposed that the variations of hip geometry changes the biomechanics of hip, leading to lower limb kinematic abnormalities and induces KOA, and malalignment which may represent the severity of disease. There is substantial literature illustrating how variations in hip geometry can alter abductor muscle forces. Weidow *et al*.^18^ revealed that increased abductor moment over the knee due to coxa valga increased the risk of lateral KOA. They analyzed women with a short functional femoral neck, and may have difficulties maintaining sufficient hip abductor strength due to a short lever arm (offset / midpelvis‐centre of the femoral head distance); thus, prompt any increase of the activity of the hip abductors that try to compensate for the small lever arm at the hip will also involve the tensor fascia lata. Since this muscle also passes through the lateral side of the knee, it plays a role in the development of forces in its lateral compartment, leading to increases in abductor moment over the knee. Additionally, if abductor moment at the knee increases, it will cause knee valgus, resulting in lateral compartment osteoarthritis. Jacobs' conclusions are consistent with the findings mentioned above. They analyzed that woman with a greater ratio of pelvic width to femur length lead the hip joint at a more adductive position, which increases the angles of the knee valgus. They showed this will increase activity of the hip abductor muscle groups in order to compensate for the above situation.^14^ Moreover, changes of the morphology of the hip joint can affect the progression of KOA by affecting hip abductor muscle strength. Therefore, we assume that if increased coxa vara, it will increase the knee adductor moment, resulting in medial compartment osteoarthritis.

We found a tendency to coxa vara in medial OA, and presence of coxa vara may be one reason for the development of medial OA; however, the relationship between these two conditions is uncertain, and few scholars have investigated this question. Some researchers have noted that the peak knee adduction moment (KAM, Indicators of KOA risk) is associated with increased progression and severity of medial KOA.^15^ They found that the reduction of the KAM indicated a shift in the knee joint load distribution from medial to lateral, whereas an increased KAM would initiate degenerative changes in the medial compartment of the knee. Due to a stance phase KAM, greater load passes medially rather than laterally even in neutral healthy knees. The KAM had a significant positive correlation with KAM.^26^, ^27^, ^28^ Foucher *et al*.^13^ researched a relationship between the hip geometry and the KAM. They found that the position of the center of the hip had the greatest impact on the load of the knee. More superior hip centers (the hip valgus is more severe) were associated with lower ipsilateral KAM and lower risk of KOA. This is consistent with our findings. Weidow *et al*.^18^ found a tendency to coxa vara in medial KOA, which reached significance only in the group with OA of the hip. However, Coskun Benlidayi *et al*.^29^ came to different conclusions. They graded the narrowing of the medial compartment of the knee joint and compared the differences in NSA between different grades. Their results showed that the narrower the medial compartment of the knee, the larger the NSA. Their conclusion was: people with an NSA greater than 134.4° have an eightfold increased risk of developing severe knee OA. However, they did not account for this result according to the leg alignment. There are some differences between the study by Coskun Benlidayi *et al*. and our study that should be mentioned. Their method of evaluating knee OA is based on the degree of the knee joint space narrowing, and they focused on severe knee OA. Whether these differences affect the test results remains to be investigated in the future. Furthermore, this also shows that coxa vara or coxa valga influence on medial knee OA or lateral knee OA may be worthy of further exploration. Pearson correlation analysis shows that the KAM was also significantly correlated with HKA. Miyazaki *et al*.^15^ found that the KAM showed a significant positive correlation with the mechanical axis (supplementary angle of the HKA). They also found the KAM is the most significant factor influencing the progression of medial knee OA. The KAM leads to increase medial knee pressure and prompts the knee varus deformity. Hurwitz *et al*.^30^ found that the single best predictor of the KAM with standing was HKA in the OA group. These studies all support our results.

### Relationship between Femoral Shaft and Knee Osteoarthritis

It remains controversial how deformity of the femoral shaft affects unicompartmental knee OA. Lasam *et al*. compared 367 women with knee OA with 60 women without knee OA. The results showed that 42.2% of women in the knee OA group had a lateral FSB >5°, and the mean lateral FSB of the OA group was greater than that of the control group. Medial knee OA may cause the bend of the femoral shaft due to varus deformity, resulting in the increase of the lateral FSB. Moreover, the increase of the lateral FSB may lead to the medial translation of the alignment of the lower limb and accelerates the degenerative change of the medial compartment of the knee joint.^31^ Matsumoto *et al*. studied 454 Japanese people with the knee OA, and reported that the coronal lateral FSB increases with age or with the severity of knee OA.^24^ Mullaji *et al*.^32^ showed that the coronal lateral FSB of patients with medial knee OA was greater than that of the healthy control group, as well as the lateral FSB of patients with knee OA whose the distal femoral axis mechanical axis angle more than 9° is greater than that of patients whose angle is <9°. Therefore the distal femoral axis mechanical axis angle and the lateral FSB showed a significant positive correlation. In our research, the coronal lateral FSB of the HTO group was greater than that of the control group. This result is consistent with the above findings. We speculate that chronic overload of the medial compartment of the knee joint may be the primary reason for the bend of the femoral shaft. In addition, during TKA, increases of the lateral FSB have been demonstrated to cause secondary knee varus deformity. During the surgical planning of the TKA or HKA, through a comparison of the lateral FSB of the full‐length X‐ray film of the lower extremity with double lower limb on CT, researchers found that greater lateral FSB increases the risk of medial knee OA.^33^, ^34^, ^35^

### Distal Femur and Knee Osteoarthritis

Micicoi *et al*. concluded that knee joints can be classified into three phenotypes based on HKA: varus, valgus, or neutral alignment. They discovered that the neutral alignment is the primary morphological pattern in the healthy middle‐aged population. In this phenotype, the distal femur was approximately in a 4° valgus (85.8° ± 2.0°) and the proximal tibia in a 4° varus (85.6° ± 2.4°). This neutrality is caused by ipsilateral femoral valgus compensating for tibial varus. There were no racial/ethnic group or sex/gender differences in this study. A CT scan‐based modeling and analysis system was used for the above research; however, the strength of the study was a large sample size (758 patients). This allowed for better extrapolation of the general results of the population.^36^ Cooke *et al*. analyzed X‐ray film to study the natural progression of knee OA and found that the initial progression of knee OA was affected by an increase in mLDFA and medial proximal tibial angle (MPTA).^37^ Analyzing 164 patients who underwent HTO for treating medial knee OA, Ahmet *et al*. found that the impact on the knee varus of the alignment of the femur as large as that of the tibia. Thus, femoral deformities should not be ignored.^38^ Lin *et al*. divided the varus knee into two types. In the first, the mean mLDFA was 88.0° ± 1.4° and the mean MPTA was 83.5° ± 1.6°, and was the major contributor to varus was varus of the tibia. The second type, the mean mLDFA was 91.4° ± 1.4°, while the mean MTPA was 85.2° ± 2.0°, and the varus of both the femur and the tibia was present. The second type was more severe in the degree of the varus knee than other types. The latter accounted for 33% of the varus types and had significantly higher mLDFA than the normal control group. These conclusions further illustrate the important effect of femoral deformity on knee varus.^39^ Thienpont *et al*. separately measured X‐ray film of the knee valgus, control, and knee varus groups, and found that the mLDFA of the varus group (89°) was significantly greater than that of the control (87°) and the valgus groups (85°).^40^ In our research, the mean mLDFA of the HTO group was significantly greater than that of the control group, this was consistent with the conclusions above. The increase of mLDFA is related to the change of NSA, because the curvature of femur leads to corresponding changes of both ends of the femur. For surgeons, during a hip replacement surgery, it is important not to create too much of a structural varus alignment by implanting the new hip joint as a varus alignment it can increase the KAM and the risk for osteoarthritis of the medial knee compartment.

### Strengths and Limitations

The advantage of our study is that it reminds us that the anatomic factors of both femur and tibia should be considered together in the treatment of medial KOA. One of the reasons why the progression of KOA did not slowdown in some patients treated with HTO alone after surgery may be that the anatomical factors of the femur were not considered. For medial KOA, the anatomical morphology of the femur and tibia is equally significant. There are some limitations to our study. On AP radiographs this variation may partly be hidden due to different rotational positions of the femur when the radiograph is exposed. Measurements of the Neck Shaft angle are subjected to methodological errors. Another limitation of the present study is that the alignment of the leg was evaluated only on the frontal plane. However, the variations of the alignment of the lower limb is often more complex. To fully understand the geometry of valgus deformity, a three‐dimensional analysis that accounts for the sagittal plane and rotation would be required and should be included in future research directions. Finally, patients were chosen based on their radiographs and not based on their clinical symptoms, as well as the definition of knee OA was Kellgren–Lawrence grade. The combination of these two methods of assessment for quantitative analysis to assess the severity of knee OA is also a direction that should be considered. Finally, generally speaking, the progression of OA is the result of the joint action of the deformity of femur and tibia. The development of OA in the HTO group could not be determined to be caused by a single femoral malformation, nor could the role of tibial malformation be ignored. But, it is obvious that the femoral malformation is definitely a risk factor for the development of medial knee OA.

## Conclusion

In conclusion, the increase of FSB, mLDFA, and varus of the hip joint can lead to medial KOA. Additionally, a combined femoral and tibial‐based deformity was more common than an isolated tibial‐based deformity. Therefore, it is necessary to evaluate the joint deformity of the femur and tibia before surgery in order to determine whether to use HTO alone to correct the lower limb alignment.

## Conflict of Interest

There are no financial conflicts of interest to disclose.

## Ethical Statement

We promise that all the data provided are true and effective, and we will carry out clinical research projects in accordance with relevant national laws and regulations such as Good Clinical Practice (GCP) and international ethical standards, as well as the requirements of the hospital and ethics committee. The study was approved by the Institutional ethics review board. If there is any conflict of interest between us and the project, we will proactively report to the Ethics Committee and the project management department and provide relevant information.

## Author Contributions

All authors had full access to the data in the study and take responsibility for the integrity of the data and the accuracy of the data analysis. Conceptualization, Ran Sun and Shiqi Qin; methodology, Ran Sun, Shiqi Qin, Ming Li; investigation, Ming Li, Yanfeng Jia, Wei Gao, Juncai Xu and Boxuan Zhang; formal Analysis, Shiqi Qin, Ming Li and Yanfeng Jia; writing—original draft, Ran Sun and Shiqi Qin; writing—review and editing, Shiqi Qin, and Ming Li; visualization, Hailong Guo and Ao Feng; supervision, Ran Sun.

## References

[1] Liu Y , Zhang H , Liang N , Fan W , Li J , Huang Z , et al. Prevalence and associated factors of knee osteoarthritis in a rural Chinese adult population: an epidemiological survey. BMC Public Health. 2016;30(16):94. 10.1186/s12889-016-2782-x PMC4736305 26830813

[2] Midgley J . Osteoarthritis and obesity; conservative management, multi‐morbidity, surgery and the implications of restricted access to knee or hip replacement: a literature review. Int J Orthop Trauma Nurs. 2021;40:100840. 33461941 10.1016/j.ijotn.2020.100840

[3] Boer CG , Hatzikotoulas K , Southam L , Stefánsdóttir L , Zhang Y , Coutinho de Almeida R , et al. Deciphering osteoarthritis genetics across 826,690 individuals from 9 populations. Cell. 2021;184(18):4784–4818. 34450027 10.1016/j.cell.2021.07.038 PMC8459317

[4] Ramazanian T , Yan S , Rouzrokh P , Wyles CC , O Byrne TJ , Taunton MJ , et al. Distribution and correlates of hip‐knee‐ankle angle in early osteoarthritis and preoperative Total knee arthroplasty patients. J Arthroplasty. 2022;37(6S):S170–S175. 35210147 10.1016/j.arth.2021.12.009 PMC9117418

[5] Colyn W , Bruckers L , Scheys L , et al. Changes in coronal knee‐alignment parameters during the osteoarthritis process in the varus knee. J ISAKOS. 2023;S2059‐7754(22):115–118. 10.1016/j.jisako.2022.12.002 36646170

[6] Mündermann A , Dyrby CO , D'Lima DD , Colwell CW Jr , Andriacchi TP . In vivo knee loading characteristics during activities of daily living as measured by an instrumented total knee replacement. J Orthop Res. 2008;26(9):1167–1172. 18404700 10.1002/jor.20655

[7] Siddiqi A , Anis H , Borukhov I , Piuzzi NS . Osseous morphological differences in knee osteoarthritis. J Bone Joint Surg Am. 2022;104(9):805–812. 35298445 10.2106/JBJS.21.00892

[8] Grammens J , Van Haver A , Danckaers F , et al. Small medial femoral condyle morphotype is associated with medial compartment degeneration and distinct morphological characteristics: a comparative pilot study. Knee Surg Sports Traumatol Arthrosc. 2021;29(6):1777–1789. 32797248 10.1007/s00167-020-06218-8 PMC8126545

[9] Suardi C , Stimolo D , Zanna L , Carulli C , Fabrizio M , Civinini R , et al. Varus morphology and its surgical implication in osteoarthritic knee and total knee arthroplasty. J Orthop Surg Res. 2022;17(1):299. 35659012 10.1186/s13018-022-03184-4 PMC9166439

[10] Moon YW , Park JH , Lee SS , Kang JW , Lee DH . Distal femoral phenotypes in Asian varus osteoarthritic knees. Knee Surg Sports Traumatol Arthrosc. 2022;30(2):456–463. 32681285 10.1007/s00167-020-06131-0

[11] Mullaji A , Shah R , Bhoskar R , Singh A , Haidermota M , Thakur H . Seven phenotypes of varus osteoarthritic knees can be identified in the coronal plane. Knee Surg Sports Traumatol Arthrosc. 2021;20:2793–2805. 10.1007/s00167-021-06676-8 34286347

[12] Kawahara S , Hara D , Murakami K , Hamai S , Akasaki Y , Tsushima H , et al. Smaller femoral neck anteversion in varus knees than in healthy and valgus knees. Clin Anat. 2022;35(8):1044–1050. 35333417 10.1002/ca.23862

[13] Foucher KC , Wimmer MA . Does hip implant positioning affect the peak external adduction moments of the healthy knees of subjects with total hip replacements? J Orthop Res. 2013;31(8):1187–1194. 23787990 10.1002/jor.22350

[14] Jacobs CA , Uhl TL , Mattacola CG , et al. Hip abductor function and lower extremity landing kinematics: sex differences. J Athl Train. 2007;42(1):76–83. 17597947 PMC1896084

[15] Miyazaki T , Wada M , Kawahara H , Sato M , Baba H , Shimada S . Dynamic load at baseline can predict radiographic disease progression in medial compartment knee osteoarthritis. Ann Rheum Dis. 2002;61(7):617–622. 12079903 10.1136/ard.61.7.617 PMC1754164

[16] Kellgren JH , Lawrence JS . Radiological assessment of osteo‐arthrosis. Ann Rheum Dis. 1957;16(4):494–502. 13498604 10.1136/ard.16.4.494 PMC1006995

[17] Akamatsu Y , Kobayashi H , Kusayama Y , Kumagai K , Saito T . Femoral shaft bowing in the coronal and sagittal planes on reconstructed computed tomography in women with medial compartment knee osteoarthritis: a comparison with radiograph and its predictive factors. Arch Orthop Trauma Surg. 2016;136(9):1227–1232. 27457723 10.1007/s00402-016-2519-4

[18] Weidow J , Mars I , Kärrholm J . Medial and lateral osteoarthritis of the knee is related to variations of hip and pelvic anatomy. Osteoarthr Cartil. 2005;13(6):471–477. 10.1016/j.joca.2005.01.009 15922181

[19] Lu Y , Zheng Z , Chen W , Lv H , Lv J , Zhang Y . Dynamic deformation of femur during medial compartment knee osteoarthritis. PloS One. 2019;14(12):e0226795. 31860687 10.1371/journal.pone.0226795 PMC6924647

[20] Kellam PJ , Rogers MJ , Myhre L , Dekeyser GJ , Maak TG , Marchand LS . Femoral neck shaft angle is not correlated with femoral version: a retrospective study of computed tomography scans. Injury. 2022;53(2):615–619. 34973830 10.1016/j.injury.2021.12.026

[21] Yau WP , Chiu KY , Tang WM , Ng TP . Coronal bowing of the femur and tibia in Chinese: its incidence and effects on total knee arthroplasty planning. J Orthop Surg (Hong Kong). 2007;15(1):32–36. 17429114 10.1177/230949900701500108

[22] Mullaji A , Bhoskar R , Singh A , Haidermota M . Valgus arthritic knees can be classified into nine phenotypes. Knee Surg Sports Traumatol Arthrosc. 2022;30(9):2895–2904. 34750671 10.1007/s00167-021-06796-1

[23] Anderson AE . CORR insights((R)): increased hip stresses resulting from a cam deformity and decreased femoral neck‐shaft angle during level walking. Clin Orthop Relat Res. 2017;475(4):1009–1012. 27785672 10.1007/s11999-016-5126-3 PMC5339135

[24] Matsumoto T , Hashimura M , Takayama K , Ishida K , Kawakami Y , Matsuzaki T , et al. A radiographic analysis of alignment of the lower extremities – initiation and progression of varus‐type knee osteoarthritis. Osteoarthr Cartil. 2015;23(2):217–223. 10.1016/j.joca.2014.11.015 25481289

[25] Razak HRBA , Micicoi G , Khakha RS , Ehlinger M , Faizan A , LiArno S , et al. Patients with varus knee osteoarthritis undergoing high tibial osteotomy exhibit more femoral varus but similar tibial morphology compared to non‐arthritic varus knees. Knee Surg Sports Traumatol Arthrosc. 2022;30(2):680–687. 33423093 10.1007/s00167-020-06426-2

[26] Zeighami A , Dumas R , Aissaoui R . Knee loading in OA subjects is correlated to flexion and adduction moments and to contact point locations. Sci Rep. 2021;11(1):8594. 33883591 10.1038/s41598-021-87978-2 PMC8060429

[27] Sharma L , Chmiel JS , Almagor O , Felson D , Guermazi A , Roemer F , et al. The role of varus and valgus alignment in the initial development of knee cartilage damage by MRI: the MOST study. Ann Rheum Dis. 2013;72(2):235–240. 22550314 10.1136/annrheumdis-2011-201070 PMC3845483

[28] D'Souza N , Charlton J , Grayson J , Kobayashi S , Hutchison L , Hunt M , et al. Are biomechanics during gait associated with the structural disease onset and progression of lower limb osteoarthritis? A systematic review and meta‐analysis. Osteoarthr Cartil. 2022;30(3):381–394. 10.1016/j.joca.2021.10.010 34757028

[29] Coskun Benlidayi I , Guzel R , Basaran S , Aksungur EH , Seydaoglu G . Is coxa Valga a predictor for the severity of knee osteoarthritis? A Cross‐Sectional Study Surg Radiol Anat. 2015;37(4):369–376. 25113012 10.1007/s00276-014-1359-6

[30] Hurwitz DE , Ryals AB , Case JP , Block JA , Andriacchi TP . The knee adduction moment during gait in subjects with knee osteoarthritis is more closely correlated with static alignment than radiographic disease severity, toe out angle and pain. J Orthop Res. 2002;20(1):101–107. 11853076 10.1016/S0736-0266(01)00081-X

[31] Lasam MP , Lee KJ , Chang CB , et al. Femoral lateral bowing and varus condylar orientation are prevalent and affect axial alignment of TKA in Koreans. Clin Orthop Relat Res. 2013;471(5):1472–1483. 23011845 10.1007/s11999-012-2618-7 PMC3613555

[32] Mullaji AB , Marawar SV , Mittal V . A comparison of coronal plane axial femoral relationships in Asian patients with varus osteoarthritic knees and healthy knees. J Arthroplasty. 2009;24(6):861–867. 18701244 10.1016/j.arth.2008.05.025

[33] Andriacchi TP , Mündermann A . The role of ambulatory mechanics in the initiation and progression of knee osteoarthritis. Curr Opin Rheumatol. 2006;18(5):514–518. 16896293 10.1097/01.bor.0000240365.16842.4e

[34] Nishida K , Matsumoto T , Takayama K , Ishida K , Nakano N , Matsushita T , et al. Remaining mild varus limb alignment leads to better clinical outcome in total knee arthroplasty for varus osteoarthritis. Knee Surg Sports Traumatol Arthrosc. 2017;25(11):3488–3494. 27506810 10.1007/s00167-016-4260-5

[35] Slevin O , Hirschmann A , Schiapparelli FF , Amsler F , Huegli RW , Hirschmann MT . Neutral alignment leads to higher knee society scores after total knee arthroplasty in preoperatively non‐varus patients: a prospective clinical study using 3D‐CT. Knee Surg Sports Traumatol Arthrosc. 2018;26(6):1602–1609. 29026941 10.1007/s00167-017-4744-y

[36] Micicoi G , Jacquet C , Sharma A , LiArno S , Faizan A , Kley K , et al. Neutral alignment resulting from tibial vara and opposite femoral valgus is the main morphologic pattern in healthy middle‐aged patients: an exploration of a 3D‐CT database. Knee Surg Sports Traumatol Arthrosc. 2021;29(3):849–858. 32372282 10.1007/s00167-020-06030-4

[37] Cooke TD , Sheehy L , Scudamore RA . What information must measures provide to demonstrate the problems in knee alignment and osteoarthritis? Osteoarthr Cartil. 2010;18(11):1544 author reply 1545. 10.1016/j.joca.2010.06.015 20713162

[38] Issın 1 A , Sahin V , Koçkara N , et al. Is proximal tibia the major problem in varus gonarthrosis? Evaluation of Femur and ankleEklem Hastalik Cerrahisi. 2012;23(3):128–133. 23145754

[39] Lin YH , Chang FS , Chen KH , Huang KC , Su KC . Mismatch between femur and tibia coronal alignment in the knee joint: classification of five lower limb types according to femoral and tibial mechanical alignment. BMC Musculoskelet Disord. 2018;19(1):411. 30474544 10.1186/s12891-018-2335-9 PMC6260902

[40] Thienpont E , Schwab PE , Cornu O , Bellemans J , Victor J . Bone morphotypes of the varus and valgus knee. Arch Orthop Trauma Surg. 2017;137(3):393–400. 28110363 10.1007/s00402-017-2626-x

